# Efficacy and adverse events of high-frequency oscillatory ventilation in adult patients with acute respiratory distress syndrome: a meta-analysis

**DOI:** 10.1186/cc13880

**Published:** 2014-05-20

**Authors:** Chun-Ta Huang, Hsien-Ho Lin, Sheng-Yuan Ruan, Meng-Sui Lee, Yi-Ju Tsai, Chong-Jen Yu

**Affiliations:** 1 Department of Internal Medicine, National Taiwan University Hospital, No. 7, Chung-Shan South Road, Taipei 100, Taiwan; 2 Department of Traumatology, National Taiwan University Hospital, No. 7, Chung-Shan South Road, Taipei 100, Taiwan; 3 Graduate Institute of Clinical Medicine, National Taiwan University, 4, Roosevelt Road, Taipei 10617, Taiwan; 4 Graduate Institute of Epidemiology and Preventive Medicine, National Taiwan University, 4, Roosevelt Road, Taipei 10617, Taiwan; 5 School of Medicine, College of Medicine, Fu-Jen Catholic University, 510, Zhongzheng Road, New Taipei 242, Taiwan

## Abstract

**Introduction:**

Theoretically, high-frequency oscillatory ventilation (HFOV) achieves all goals of a lung-protective ventilatory mode and seems ideal for the treatment of adult patients with acute respiratory distress syndrome (ARDS). However, its effects on mortality and adverse clinical outcomes remain uncertain given the paucity of high-quality studies in this area. This meta-analysis was performed to evaluate the efficacy and adverse events of HFOV in adults with ARDS.

**Methods:**

We searched PubMed, EMBASE and Cochrane Central Register of Controlled Trials through February 2014 to retrieve randomized controlled trials of HFOV in adult ARDS patients. Two independent reviewers extracted data on study methods, clinical and physiological outcomes and adverse events. The primary outcome was 30-day or hospital mortality. Risk of bias was evaluated with the Cochrane Collaboration’s tool. Mortality, oxygenation and adverse effects of HFOV were compared to those of conventional mechanical ventilation. A random-effects model was applied for meta-analysis.

**Results:**

A total of five trials randomly assigning 1,580 patients met inclusion criteria. Pooled data showed that HFOV significantly improved oxygenation on day one of therapy (four studies; 24% higher; 95% confidence interval (CI) 11 to 40%; *P* <0.01). However, HFOV did not reduce mortality risk (five studies; risk ratio (RR) 1.04; 95% CI 0.83 to 1.31; *P* = 0.71) and two early terminated studies suggested a harmful effect of HFOV in ARDS (two studies; RR 1.33; 95% CI 1.09 to 1.62; *P* <0.01). Safety profiles showed that HFOV was associated with a trend toward increased risk of barotrauma (five studies; RR 1.19; 95% CI 0.83 to 1.72; *P* = 0.34) and unfavorable hemodynamics (five studies; RR 1.16; 95% CI 0.97 to 1.39; *P* = 0.12).

**Conclusions:**

HFOV improved oxygenation in adult patients with ARDS; however, it did not confer a survival benefit and might cause harm in the era of lung-protective ventilation strategy. The evidence suggests that HFOV should not be a routine practice in ARDS and further studies specifically selecting patients for this ventilator mode should be pursued.

## Introduction

Acute respiratory distress syndrome (ARDS) is a syndrome resulting from acute, diffuse, inflammatory lung injury and is associated with increased pulmonary vascular permeability, increased lung weight and loss of aerated tissue [1]. It is associated with a variety of systemic and pulmonary insults, and clinically characterized by acute onset of respiratory failure associated with hypoxemia refractory to oxygen therapy and bilateral radiographical opacities. ARDS is the most severe form of lung injury and carries an appreciable mortality rate [1].

Conventional mechanical ventilation (CMV) remains the cornerstone of therapy for ARDS patients; however, mechanical ventilation per se may worsen a preexisting lung injury through overdistension of alveoli and cyclic atelectasis, and produce ventilator-induced lung injury [2]. In 2000, a landmark trial demonstrated that mechanical ventilation with a lower tidal volume (6 ml/kg) than was traditionally used (12 ml/kg) results in decreased mortality in patients with ARDS [3], and the observed benefit is probably explained by reduction of ventilator-induced lung injury. From then on, a lung-protective ventilation strategy has been widely adopted for the management of ARDS. However, despite progress in critical care and our better understanding of the pathophysiological mechanisms responsible for ARDS, its mortality remains as high as 48% [4].

High-frequency oscillatory ventilation (HFOV), developed by Lunkenheimer *et al*. in 1972 [5], delivers very small tidal volumes (1 to 4 ml/kg) at a frequency range of 3 to 15 Hz while maintaining a high mean airway pressure. The evidence from observational studies showed that HFOV could improve oxygenation when employed as a rescue therapy after failing CMV in patients with ARDS [6-11]. HFOV is a theoretically ideal lung-protective ventilation mode to prevent development of ventilator-induced lung injury by limiting excess alveolar distension and achieving greater lung recruitment. However, previous clinical trials failed to provide convincing evidence to prove the efficacy of HFOV in adult patients with ARDS due to small sample size [12-14]. In addition, uncertainty exists regarding overall evidence for adverse effects of HFOV, such as barotrauma and hemodynamic compromise [15,16]. Two large randomized controlled trials of HFOV in ARDS had published their results in early 2013 [17,18]. It is anticipated that an updated meta-analysis may help clarify the role of HFOV in adult ARDS.

In this study, we conducted a systematic review and meta-analysis to evaluate the efficacy of HFOV in terms of oxygenation and mortality and the adverse events associated with the use of HFOV.

## Materials and methods

The meta-analysis was conducted following the PRISMA guideline [19], and the study protocol has been registered in and approved by the PROSPERO International prospective register of systematic reviews (CRD42013005065). No research ethics committee approval or patient consents were required for this meta-analysis because it evaluated published studies.

### Identification of studies

We searched PubMed, EMBASE and the Cochrane Central Register of Controlled Trials (up to February 2014) using predefined strategies (Additional file 1). We also hand searched references from included studies and review articles, and conference proceedings of the American College of Chest Physicians (2003 to 2013), American Thoracic Society (2004 to 2013), Society of Critical Care Medicine (1995 to 2014), European Society of Intensive Care Medicine (1995 to 2013), British Thoracic Society (2005 to 2013) and European Respiratory Society (2001 to 2013). Moreover, we searched for ongoing or unpublished trials in trial registry websites (clinicaltrials.gov and controlled-trials.com). No language restrictions were applied.

### Study eligibility

Retrieved studies were independently evaluated for possible inclusion in this review by two reviewers (SYR, MSL) and disagreement was resolved by consensus. Inclusion criteria were study design in randomized controlled trial, study population of ARDS defined according to the American-European Consensus Conference definition [20], and the use of HFOV as front-line therapy in one study arm. Exclusion criteria included study population of pediatric or neonatal patients and another concomitant intervention for ARDS, such as inhaled nitric oxide or prone position.

### Data extraction and quality assessment

Two reviewers (SYR, MSL) independently extracted data from included studies. When necessary, we contacted with the authors for more details of their studies and gathered information from prior reviews [15,16,21]. We extracted the demographics and types of participants, disease severity assessed by partial pressure of oxygen in arterial blood/fraction of inspired oxygen (PaO_2_/F_I_O_2_) and Acute Physiology and Chronic Health Evaluation II (APACHE II), ventilator days before enrollment, ventilator-associated parameters, and concomitant interventions. We evaluated the quality of the studies using the Cochrane Collaboration’s tool for assessing risk of bias [22].

### Outcome measures

The primary outcome was hospital mortality or 30-day mortality as an alternative [23]. Secondary outcomes included intensive care unit (ICU) mortality, duration of mechanical ventilation, ventilator-free days to 30 days, gas exchange (PaO_2_/F_I_O_2_ and partial pressure of carbon dioxide in arterial blood (PaCO_2_)) and adverse events (barotrauma and unfavorable hemodynamics).

### Statistical analyses

Statistical analyses were performed using Stata version 11 (StataCorp, College Station, TX, USA). Continuous variables were analyzed using weighted mean differences (WMD) or ratios of means [24] with their 95% confidence intervals (CIs). Binary outcomes were pooled by risk ratios (RRs). A random-effects model was applied for all analyses. Heterogeneity between studies was evaluated with the Cochran Q test and I^2^, and statistical heterogeneity was considered low if I^2^ was 25 to 49%, moderate if I^2^ was 50 to 74% and high if I^2^ was ≧75% [25]. A funnel plot and the Egger’s test were used to evaluate publication bias. Prespecified subgroup analysis was performed to assess the influence of adherence to a lung-protective ventilation strategy in the CMV arm on the primary outcome. Studies were considered to adopt a lung-protective ventilation strategy if they targeted a tidal volume of 6 to 8 ml/kg in the CMV group. Furthermore, to evaluate whether the tidal volume was a significant source of between-study heterogeneity, meta-regression analysis was used to search for a dose-response relationship between mortality (log of RR) and mean tidal volume per ideal body weight (ml/kg) in the CMV arm. Statistical significance was set at the two-sided 0.05 level for hypothesis testing and 0.1 for heterogeneity testing.

## Results

### Study flow and characteristics

**Figure 1:**
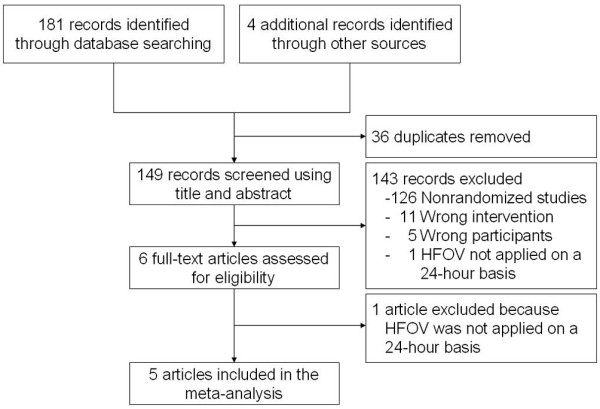
**Flow diagram of the review.** HFOV, high-frequency oscillatory ventilation.

We identified 181 articles from searches of electronic databases and four articles from other sources (Figure 1). Full-text assessment was carried out for six potentially eligible papers and one [26] of them was excluded given that HFOV was not provided on a 24-hour basis (Additional file 2). Finally, five studies [12-14,17,18] were included for narrative synthesis and meta-analysis.

Table 1 describes characteristics of included studies. Five studies [12-14,17,18] enrolled 1,580 patients, of whom 800 were ventilated with HFOV. Patients were included in the study shortly after institution of mechanical ventilation (≦a mean of 3.5 [12,14,17,18] or <5 [13] days). The median APACHE II was 21.8 (range, 19.0 to 29). The HFOV settings varied among studies (Table 2), and different adjustment protocols were employed. In the CMV group, patients were placed on pressure control ventilation in all studies, and three [13,17,18] of these adopted a lung-protective ventilation strategy. Positive end-expiratory pressure was adjusted according to the ARDS Network protocol [3] or Lung Open Ventilation Study [27] in two [13,17] and one [18] studies, respectively. Patients ventilated with CMV in the study by Ferguson *et al*. [18] received the smallest tidal volume per ideal body weight on study entry.

**Table 1:** Characteristics of the five adult studies included in the review

	**Derdak *et al*. 2002 **[12]	**Shah *et al*. 2004 **[13]	**Bollen *et al*. 2005 **[14]	**Ferguson *et al*. 2013 **[18]	**Young *et al*. 2013 **[17]
Patients, number	148	28	61	548	795
Mean age, year	49.5	49.2	52.5	54.5	55.4
Male sex, number (%)	86 (58%)	18 (64%)	42 (69%)	320 (58%)	495 (62%)
APACHE II, mean^a^	22	19.0	20.7	29	21.8
Enrollment period	1997-2000	Not reported	1997-2001	2007-2012	2007-2012
Enrollment criteria	PaO_2_/F_I_O_2_ ≦200 with PEEP ≧10 cmH_2_O	ARDS^b^	PaO_2_/F_I_O_2_ < 200	PaO_2_/F_I_O_2_ ≦200 with F_I_O_2_ ≧0.5	PaO_2_/F_I_O_2_ ≦200 with PEEP ≧5 cmH_2_O
Mean PaO_2_/F_I_O_2_ at enrollment	112.5	110.6	22.4^c^	117.5	113
Ventilator days prior to study	3.5 (mean)	<5	1.9 (mean)	2.2 (mean)	2.2 (mean)

^a^Scored at randomization, except the study by Ferguson *et al*., in which scored at admission to the intensive care unit; ^b^based on the American-European Consensus Conference definition of the acute respiratory distress syndrome; ^c^oxygenation index. APACHE II, Acute Physiology and Chronic Health Evaluation II; PaO_2_, partial pressure of oxygen in arterial blood; F_I_O_2_, fraction of inspired oxygen; PEEP, positive end-expiratory pressure; ARDS, acute respiratory distress syndrome.

**Table 2:** Ventilator settings in included studies

	**Derdak *et al*. 2002 **[12]	**Shah *et al*. 2004 **[13]	**Bollen *et al*. 2005 **[14]	**Ferguson *et al*. 2013 **[18]	**Young *et al*. 2013 **[17]
HFOV					
Mean airway pressure	5 cmH_2_O above mean airway pressure on CMV	5 cmH_2_O above mean airway pressure on CMV	5 cmH_2_O above mean airway pressure on CMV	30 cmH_2_O	5 cmH_2_O above plateau pressure on CMV
Frequency, Hz	5	5	5	3-12	10
Amplitude	To achieve chest wall vibration to the level of the midthigh	To achieve chest wall to midthigh vibration	According to PaCO_2_ and to achieve chest wall vibration	90 cmH_2_O	A cycle volume of 100 ml
CMV					
Mode	Pressure control ventilation	Pressure control ventilation	Pressure control ventilation	Pressure control ventilation	Pressure control ventilation
Tidal volume	6-10 ml/kg (actual body weight)	A mean of 7-8 ml/kg (ideal body weight)	A mean of 8-9 ml/kg (ideal body weight)	6 ml/kg (predicted body weight)	6-8 ml/kg (ideal body weight)
Adjustment of PEEP	Study protocol	ARDS Network protocol	Not reported	Lung Open Ventilation Study	ARDS Network protocol

HFOV, high-frequency oscillatory ventilation; CMV, conventional mechanical ventilation; PaCO_2_, partial pressure of carbon dioxide in arterial blood; PEEP, positive end-expiratory pressure; ARDS, acute respiratory distress syndrome.

### Methodological quality

All studies used adequate methods of sequence generation and had adequate concealment of allocations prior to assignment. Participants, key study personnel and outcome assessors were not blinded due to the type of intervention. There was complete follow-up in all studies except one [14] for the primary outcome. In that study, follow-up time to 30 days was incomplete in 7 out of 61 patients [14]. Two studies [12,14] received financial support from commercial companies. One study [14] was stopped early due to slow enrollment and completion of a similar study, and the other [18] was prematurely terminated to act on a unanimous recommendation from the data monitoring committee. All studies reported unplanned crossovers between groups and these involved <5% of the randomized patients. In short, despite having a few flaws, three studies [12,13,17] were classified as low risk of bias, and the others [14,18] were classified as unclear risk of bias (Table 3).

**Table 3:** Risk of bias assessment

	**Derdak *et al*. 2002 **[12]	**Shah *et al*. 2004 **[13]	**Bollen *et al*. 2005 **[14]	**Ferguson *et al. *** **2013**[18]	**Young *et al*. 2013**[17]
Random sequence generation	Low	Low	Low	Low	Low
Allocation concealment	Low	Low	Low	Low	Low
Incomplete outcome data	Low	Low	Unclear	Low	Low
Selective reporting	Low	Low	Low	Low	Low
Other sources of bias					
Unplanned crossovers <5%	Low	Low	Low	Low	Low
Premature termination of trial	Low	Low	Unclear	Unclear	Low
Overall risk of bias	Low	Low	Unclear	Unclear	Low

### Data synthesis

#### Mortality

**Figure 2:**
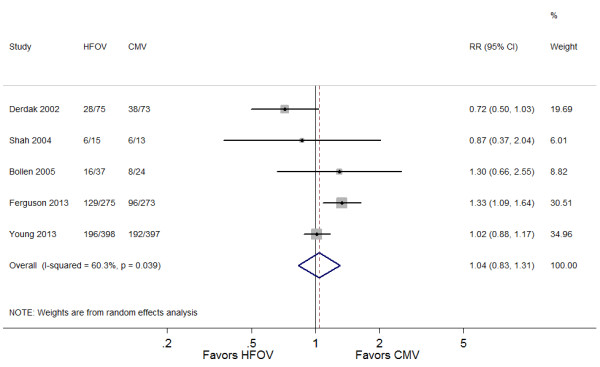
**Forest plot showing the effect of HFOV on 30-day or hospital mortality.** HFOV, high-frequency oscillatory ventilation; CMV, conventional mechanical ventilation; RR, risk ratio; CI, confidence interval.

**Figure 3:**
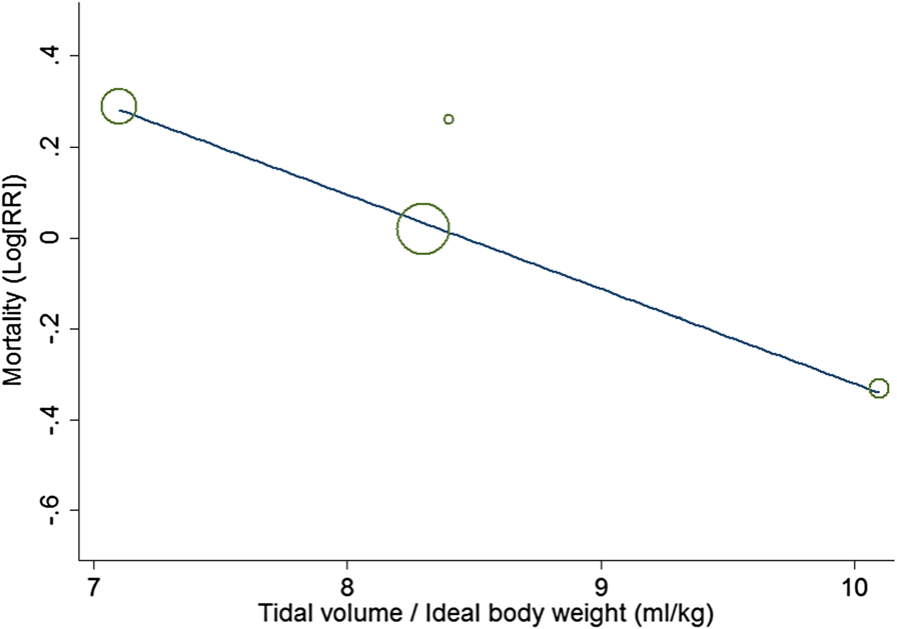
Scatter plot depicting the log of risk ratio (Log(RR)) for mortality according to the true tidal volume per ideal body weight of patients with conventional mechanical ventilation in a meta-regression analysis of four randomized controlled trials of high-frequency oscillatory ventilation in the treatment of acute respiratory distress syndrome.

There was no significant difference in hospital or 30-day mortality for patients treated with HFOV versus CMV (RR 1.04; 95% CI 0.83 to 1.31; *P* = 0.71) (Figure 2). The heterogeneity among studies was moderate (I^2^ = 60%; *P* = 0.04). Meta-regression analysis showed that tidal volume per ideal body weight in the CMV group was negatively associated with RR of mortality (Figure 3) and accounted for most of the between-study heterogeneity (adjusted R^2^ = 100%). In three studies [13,17,18], in which a lung-protective ventilation strategy was adopted (Additional file 3), HFOV seemed to increase mortality risk of ARDS patients (RR 1.13; 95% CI 0.90 to 1.42; *P* = 0.30).

Two studies [17,18] reported ICU mortality, and pooled results did not favor the use of HFOV in ARDS patients (RR 1.23; 95% CI 0.89 to 1.68; *P* = 0.21). In addition, HFOV significantly increased mortality in ARDS in two early terminated studies (RR 1.33; 95% CI 1.09 to 1.62; *P* <0.01) (Additional file 4) [14,18]. The funnel plot (Additional file 5) and the Egger’s test (*P* = 0.79) suggested no significant publication bias.

#### Ventilator days and ventilator-free days

Data about the mean and standard deviation of duration of mechanical ventilation were provided in two studies [12,17]. There was no significant difference in length of ventilator days between HFOV and CMV groups (WMD 0.85 days; 95% CI -0.96 to 2.67 days; *P* = 0.36). Another two studies [14,18] reporting the median and range also found no difference in ventilator days. Only one study [17] provided information with regard to ventilator-free days in patients with ARDS. In this study, ventilator-free days by day 30 did not differ between patients treated with HFOV or CMV (17.1 ± 8.6 versus 17.6 ± 8.8 days; *P* =0.42).

#### Physiological outcomes

Table 4 displays physiological outcomes of patients with ARDS on days 1 to 3 after study commencement. On the first day of therapy, HFOV was associated with improvement in the PaO_2_/F_I_O_2_ (four studies; 24% higher; 95% CI 11 to 40%; *P* <0.01) and the treatment effect sustained to study day 3. The mean airway pressure was significantly higher by 25 to 31% in the HFOV treated groups through day 1 to day 3. Differences in PaCO_2_ were not significant on any day.

**Table 4:** Effects of high-frequency oscillatory ventilation on physiological outcomes in patients with acute respiratory distress syndrome

**Outcome**	**Number of studies (patients)**	**Ratio of means (95% CI); *P *** **value**	***P *** **value for heterogeneity; I**^**2**^
PaO_2_/F_I_O_2_			
Day 1	4 (1032)	1.24 (1.11-1.40); <0.001	0.119; 49%
Day 2	4 (1032)	1.13 (0.94-1.37); 0.2	0.002; 79%
Day 3	4 (1032)	1.16 (0.99-1.35); 0.072	0.012; 73%
Mean airway pressure, cmH_2_O			
Day 1	4 (785)	1.31 (1.25-1.36); <0.001	0.001; 81%
Day 2	3 (237)	1.28 (1.18-1.38); <0.001	0.002; 84%
Day 3	4 (785)	1.25 (1.15-1.36); <0.001	<0.001; 90%
PaCO_2_, mmH_2_O			
Day 1	5 (1580)	0.97 (0.87-1.07); 0.494	<0.001; 94%
Day 2	4 (1032)	0.96 (0.83-1.11); 0.554	<0.001; 97%
Day 3	5 (1580)	1.11 (0.99-1.23); 0.068	<0.001; 96%

CI, confidence interval; PaO_2_, partial pressure of oxygen in arterial blood; F_I_O_2_, fraction of inspired oxygen; PaCO_2_, partial pressure of carbon dioxide in arterial blood.

#### Adverse effects

**Figure 4:**
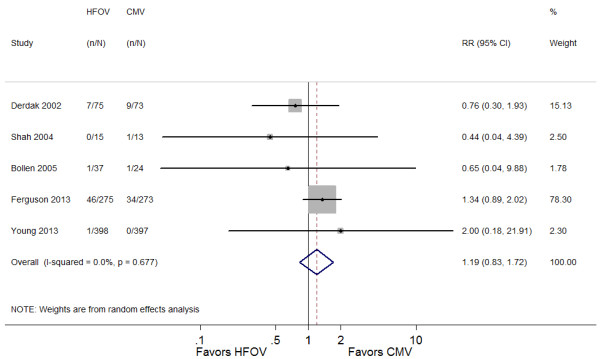
**Forest plot comparing barotrauma effects of HFOV and CMV.** HFOV, high-frequency oscillatory ventilation; CMV, conventional mechanical ventilation; RR, risk ratio; CI, confidence interval.

**Figure 5:**
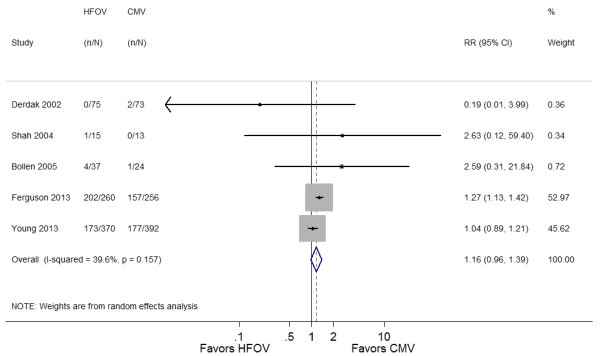
**Forest plot comparing hemodynamic effects of HFOV and CMV.** HFOV, high-frequency oscillatory ventilation; CMV, conventional mechanical ventilation; RR, risk ratio; CI, confidence interval.

Table 5 provides details of adverse effects. The application of HFOV was associated with a trend toward increased risk of barotrauma (RR 1.19; 95% CI 0.83 to 1.72; *P* = 0.34) (Figure 4). In addition, unfavorable hemodynamics were more likely to be observed in patients placed on HFOV (RR 1.16; 95% CI 0.97 to 1.39; *P* = 0.12) (Figure 5).

**Table 5:** Adverse effects of included studies

Derdak *et al*. 2002 [12]	Barotrauma. Air leak developed or worsened: HFOV 7/75, CMV 9/73.
	Hypotension. Intractable hypotension: HFOV 0/75, CMV 2/73.
	Endotracheal tube obstruction: HFOV 4/75, CMV 3/73.
Shah *et al*. 2004 [13]	Barotrauma: HFOV 0/15, CMV 1/13.
	Hypotension: HFOV 1/15, CMV 0/13.
Bollen *et al*. 2005 [14]	Barotrauma. Air leak (therapy failure): HFOV 1/37, CMV 1/24.
	Hypotension (therapy failure): HFOV 4/37, CMV 1/24.
Ferguson *et al*. 2013 [18]	Barotrauma. New-onset barotrauma: HFOV 46/256, CMV 34/259^a^.
	Hypotension. Vasopressor on study day 1: HFOV 202/260, CMV 157/256.
Young *et al*. 2013 [17]	Barotrauma. Reported as serious adverse events: HFOV 1/398, CMV 0/397.
	Hypotension. Vasoactive or inotropic agent on study day 1: HFOV 173/370, CMV 177/392.

^a^Excluding patients who had barotraumas at baseline. HFOV, high-frequency oscillatory ventilation; CMV, conventional mechanical ventilation.

## Discussion

Our meta-analysis includes five randomized controlled trials that evaluated the efficacy of HFOV as compared with CMV on mortality and improvement of oxygenation in ARDS. Overall, HFOV did improve oxygenation but it did not reduce mortality in ARDS patients. In studies that were stopped prematurely or adopted a lung-protective ventilation strategy, increased risk of mortality risk was observed in HFOV-ventilated patients. In addition, the application of HFOV was associated with a trend toward increased risk of barotrauma and unfavorable hemodynamics. Current evidence did not support the routine use of HFOV for ARDS patients in the era of lung-protective ventilation because of its potential harm.

Our study updated the data of HFOV in adult ARDS and there were some disparities between previous meta-analyses and ours [15,16]. The disparities come from exclusion of pediatric studies and inclusion of two large-scale studies. Pediatric patients have different respiratory physiology and mechanics from those of adult patients [28,29]. The patient number of the two additional studies [17,18] outnumbered the patients in prior meta-analyses. Furthermore, our study evaluated the heterogeneity brought about by the variability of tidal volumes. Meta-regression revealed a trend toward higher mortality with HFOV in the setting that smaller tidal volumes were adopted in the CMV group. The findings suggest that HFOV did not bring additional benefit once the lung-protective ventilation strategy had been strictly applied. In addition, our analysis showed that the application of HFOV was associated with potential harm. Pooled data in this meta-analysis suggest a trend toward increased risk of barotrauma and unfavorable hemodynamics in patients placed on HFOV. These adverse effects may be mediated by high airway and intrathoracic pressure. Higher mean airway pressure not only decreases venous return or increases right ventricular afterload, leading to hemodynamic compromise [30], but probably increases the risk of barotrauma [31]. These complications may partly explain the unfavorable mortality outcomes for HFOV.

Our results demonstrate statistically significant improvement in oxygenation during the early period of randomization, yet this did not translate into a clinical benefit. There are several possibilities. First, transient improvement in oxygenation may be unable to confer any survival benefit [3], in that oxygenation improvement does not necessarily indicate reduced severity of lung injury or resolution of the etiology of ARDS. Second, most ARDS patients succumb to multiple organ dysfunction syndrome rather than hypoxemia per se [32]. Third, the advantage of improved oxygenation is probably offset by a harmful HFOV strategy, which perpetuated hemodynamic instability [30]. Finally, the negative effect of barotrauma brought about by HFOV may be influential on the potential benefit of improvement in oxygenation [18].

Despite the published literature, the optimal ventilator setting for HFOV in adults remains unclear. The recruitability in ARDS patients is quite heterogeneous and unpredictable [33]; thus, it is crucial but complex to achieve adequate mean airway pressure. High mean airway pressure leads to alveolar overdistension and low mean airway pressure may fail to resolve atelectasis. Both are potentially detrimental to the patients. Additionally, we should utilize the highest possible frequency of HFOV in ARDS because the higher frequencies, the more easily a collapsed lung could be opened, leading to more homogeneous distribution of volumes and reduction in lung injury [34-36]. Finally, the resonance phenomenon may take place during HFOV [37], and some study found that the delivered gas volume actually exceeded the stroke volume at certain frequencies [37]. Therefore, knowledge of the true tidal volume is critical and helpful in tailoring HFOV settings. Currently used respiratory parameters are of limited value in providing point-of-care fine-tuning of HFOV; new monitoring technologies should be incorporated into future HFOV protocols in the hope of optimizing the efficacy of the machine [38].

The pooled RR of the early terminated studies suggests a deleterious effect of HFOV on ARDS patients. Although studies that are stopped prematurely on the basis of harm typically exaggerate the magnitude of the effect [39], the findings raise serious concerns about the unselected and widespread use of HFOV for management of adult patients with ARDS. Like other interventions for ARDS, such as inhaled nitric oxide and prone ventilation, which may not confer benefits on all ARDS patients [40,41], proper patient selection may be of paramount importance regarding the application of HFOV in ARDS. Perhaps ARDS patients require personalized therapy that takes into consideration the underlying cause and mechanism of ARDS, disease severity, cardiopulmonary responses, and co-interventions.

One of the strengths of this meta-analysis is to increase statistical power over individual studies and explore the heterogeneity between studies. We used a comprehensive search strategy to ensure that all relevant sources of evidence have been identified, and followed a prespecified protocol to avoid issues of *post hoc* analyses and *ad hoc* selection of outcomes. Additionally, we applied several methods to reduce bias, such as analyses of various relevant clinical and physiological outcomes and overall methodological bias assessment.

Although there is a moderate degree of between-study heterogeneity for the primary outcome in this meta-analysis and we demonstrate that differences in the actual tidal volume delivered to CMV-ventilated patients may statistically explain the majority of the heterogeneity, other contributing factors to heterogeneity remain worth consideration. A remarkable variation in disease severity was observed across study populations. In particular, the APACHE II score seemed to be higher in the study by Ferguson *et al*. [18], even if we consider that the score was calculated at a different time point (at ICU admission rather than at randomization). Also, variations in ventilatory strategies of HFOV were evident between studies. The results of the meta-analysis should, therefore, be interpreted with caution. However, this study provides the best available evidence about the efficacy of HFOV in adults with ARDS and discloses the impact of tidal volume in CMV on the outcome of HFOV trials. Future studies on this field would take advantage of this meta-analysis to design more appropriate trials on HFOV in adult ARDS patients.

Our study has potential limitations. First, although we did not find evidence of publication bias, the visual inspection of the funnel plot was unreliable and the test for publication bias was underpowered given the small number of studies contributing to the analysis of the main outcome. Similarly, subgroup analysis and meta-regression might also be underpowered to detect significant effects. Second, we only retrieved data from randomized controlled trials. Yet we meant to do it this way because randomized controlled trials are considered to provide the most methodologically rigorous evidence.

## Conclusions

This meta-analysis shows a beneficial effect of HFOV on oxygenation in patients with ARDS. Despite improvement in oxygenation, current available evidence suggests that HFOV does not confer a survival benefit and possibly increases mortality and complications in the era of lung-protective ventilation strategy. Thus, our findings do not justify the use of HFOV as a standard intervention for ARDS. For now, clinicians may still consider HFOV for life-threatening hypoxemia, in conjunction with other supportive therapy, based on their judgment and empirical evidence. In future works, proper patient selection and pursuit of optimal HFOV settings are two main goals for researchers interested in respiratory care.

## Key messages

• The best evidence to date does not justify the routine application of HFOV as front-line therapy in adult ARDS; instead, physicians should take patients’ conditions into account and prescribe HFOV with more caution for patients with persistent life-threatening hypoxemia despite conventional mechanical ventilation and a lung-protective ventilation strategy.

• Future studies on HFOV need to identify proper candidates who may benefit most from HFOV and to determine the optimal oscillator settings.

## Abbreviations

APACHE II: Acute Physiology and Chronic Health Evaluation II; ARDS: acute respiratory distress syndrome; CI: confidence interval; CMV: conventional mechanical ventilation; F_I_O_2_: fraction of inspired oxygen; HFOV: high-frequency oscillatory ventilation; ICU: intensive care unit; PaCO_2_: partial pressure of carbon dioxide in arterial blood; PaO_2_: partial pressure of oxygen in arterial blood; RR: risk ratio; WMD: weighted mean differences.

## Competing interests

The authors declare that they have no competing interests.

## Authors’ contributions

CTH, HHL and CJY contributed to study design. CTH, HHL, SYR, MSL and YJT contributed to study conduct. CTH, HHL, SYR and MSL contributed to manuscript writing. YJT and CJY contributed to revision of the manuscript. All authors approved the version to be published.

## Supplementary Material

Additional file 1 Search strategies. Click here for file

Additional file 2 Characteristics of the excluded study. Click here for file

Additional file 3 **Forest plot showing subgroup analysis for 30-day or hospital mortality comparing studies adopting or not adopting the lung-protective ventilation strategy.** HFOV, high-frequency oscillatory ventilation; CMV, conventional mechanical ventilation; RR, risk ratio; CI, confidence interval. Click here for file

Additional file 4 **Forest plot showing sensitivity analysis for 30-day or hospital mortality comparing studies with and without early stopping.** HFOV, high-frequency oscillatory ventilation; CMV, conventional mechanical ventilation; RR, risk ratio; CI, confidence interval. Click here for file

Additional file 5 **Funnel plot showing the effect estimates (Log(RR)) by their standard errors (SE of Log(RR)).** RR, risk ratio. Click here for file
