# Supplementary material for: Efficacy and adverse events of high-frequency oscillatory ventilation in adult patients with acute respiratory distress syndrome: a meta-analysis
Source: Crit Care. 2014 May 20;18(3):R102. doi: 10.1186/cc13880 (PMC4075239; doi:10.1186/cc13880)
Supplement: Additional file 1 — Search strategies. [file cc13880-S1.doc]

**Search strategies**

**PubMed**

("high-frequency ventilation"[MeSH Terms]) AND ("respiratory distress syndrome, adult"[MeSH Terms]) AND ("humans"[MeSH Terms]) NOT ("perinatal"[Title/abstract] OR "neonatal"[Title/abstract] OR "infant"[Title/abstract] OR "infants"[Title/abstract] OR "child"[Title/abstract] OR "children"[Title/abstract] OR "adolescent"[Title/abstract] OR "adolescents"[Title/abstract]) AND (random* OR controlled trial OR clinical trial OR randomized controlled trial OR placebo OR double-blind)

**EMBASE**

('high frequency ventilation'/exp OR high NEAR/3 oscillat*) AND 'adult respiratory distress syndrome'/exp AND 'article'/it AND 'human'/de NOT ('case report'/de OR 'case study'/de OR 'observational study'/de OR 'retrospective study'/de)

**Cochrane Central Register of Controlled Trials**

"high-frequency ventilation"[MeSH Terms] AND "respiratory distress syndrome, adult"[MeSH Terms]
