# Supplementary material for: Efficacy and adverse events of high-frequency oscillatory ventilation in adult patients with acute respiratory distress syndrome: a meta-analysis
Source: Crit Care. 2014 May 20;18(3):R102. doi: 10.1186/cc13880 (PMC4075239; doi:10.1186/cc13880)
Supplement: Additional file 2 — Characteristics of the excluded study. [file cc13880-S2.doc]

**Characteristics of the excluded study**

| Study | Reason for exclusion |
| --- | --- |
| Mentzelopoulos et al.[1] | Patients in the study received HFOV as a recruitment maneuver. Moreover, HFOV sessions were interspersed with lung-protective CMV. |

1. Mentzelopoulos SD, Malachias S, Zintzaras E, Kokkoris S, Zakynthinos E, Makris D, Magira E, Markaki V, Roussos C, Zakynthinos SG: **Intermittent recruitment with high-frequency oscillation/tracheal gas insufflation in acute respiratory distress syndrome**. *Eur Respir J* 2012, **39**:635-647.
