# Supplementary figures and images for: Efficacy and adverse events of high-frequency oscillatory ventilation in adult patients with acute respiratory distress syndrome: a meta-analysis
Source: Crit Care. 2014 May 20;18(3):R102. doi: 10.1186/cc13880 (PMC4075239; doi:10.1186/cc13880)

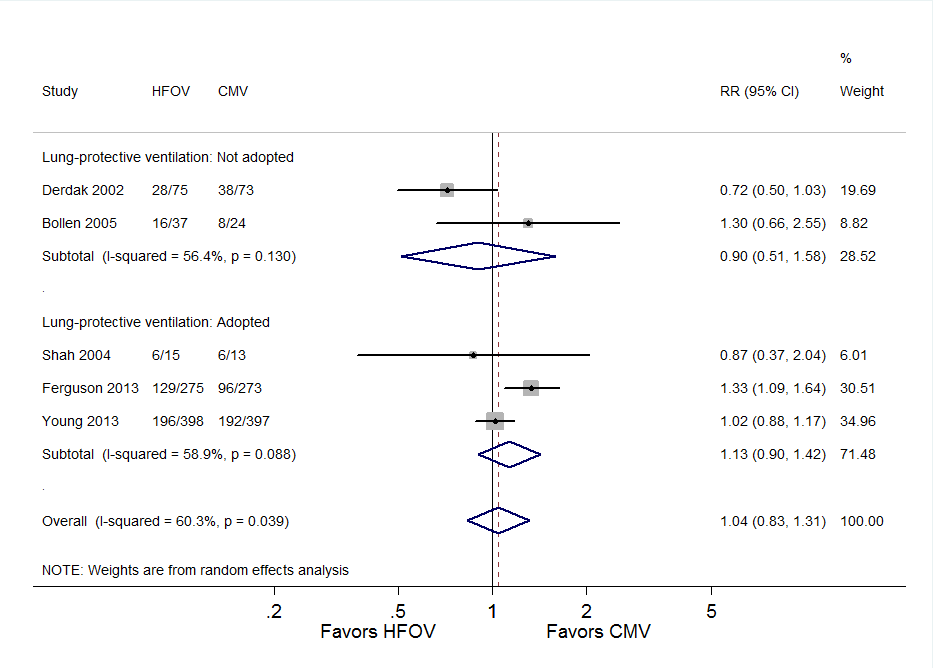

Supplement: Additional file 3 — Forest plot showing subgroup analysis for 30-day or hospital mortality comparing studies adopting or not adopting the lung-protective ventilation strategy. HFOV, high-frequency oscillatory ventilation; CMV, conventional mechanical ventilation; RR, risk ratio; CI, confidence interval. [file cc13880-S3.tiff]

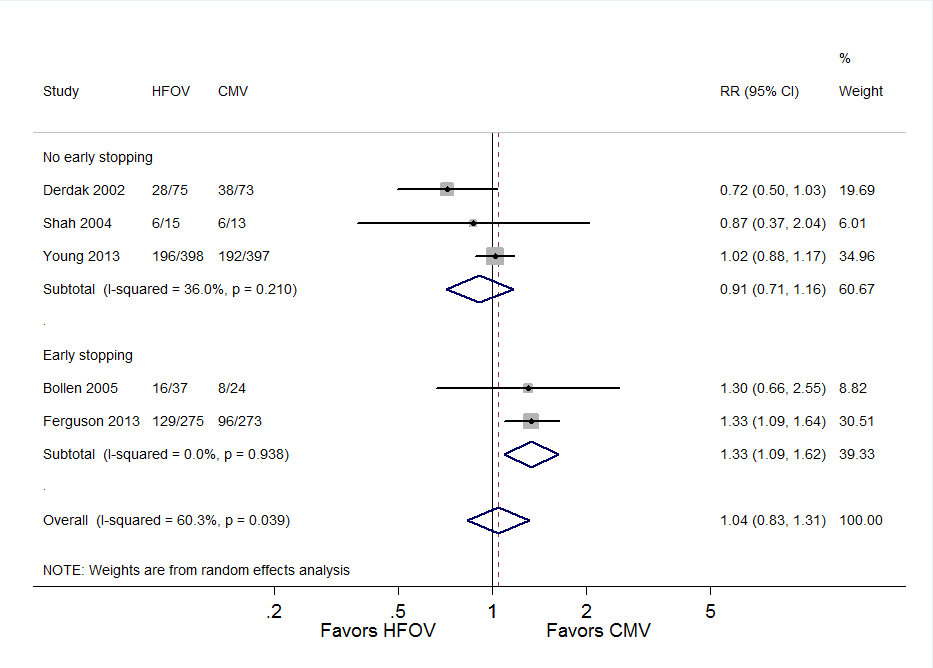

Supplement: Additional file 4 — Forest plot showing sensitivity analysis for 30-day or hospital mortality comparing studies with and without early stopping. HFOV, high-frequency oscillatory ventilation; CMV, conventional mechanical ventilation; RR, risk ratio; CI, confidence interval. [file cc13880-S4.tiff]

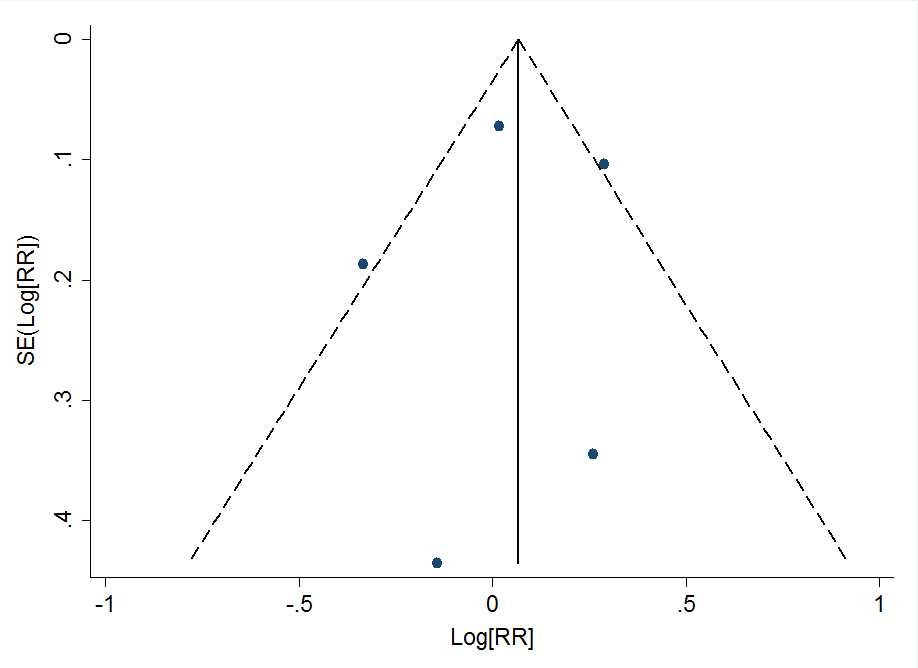

Supplement: Additional file 5 — Funnel plot showing the effect estimates (Log(RR)) by their standard errors (SE of Log(RR)). RR, risk ratio. [file cc13880-S5.tiff]
